# Effects of Differences in Fibre Composition and Maturity of Forage-Based Diets on the Fluid Balance, Water-Holding Capacity and Viscosity in Equine Caecum and Colon Digesta

**DOI:** 10.3390/ani12233340

**Published:** 2022-11-29

**Authors:** Sara Muhonen, Christelle Philippeau, Véronique Julliand

**Affiliations:** 1 L’Institut Agro Dijon, Burgundy-Franche-Comté University, PAM UMR A 02.102, 21000 Dijon, France; 2 L’Institut Agro Dijon, 26 Boulevard Dr. Petitjean, BP87999, 21079 Dijon, France

**Keywords:** forage fibre, hindgut, grass, lucerne, fluid reservoir

## Abstract

**Simple Summary:**

Horses are large herbivorous hindgut fermenters, and the equine hindgut has been suggested to function as a fluid reservoir when dietary fibre holds water in the intestinal content. Forage-based diets and plant-fibre contributes the most to this hindgut fluid reservoir. However, the fibre content and fibre composition of forage can differ greatly and there is limited research published on which fibre source and what type of fibre that would be the most appropriate. We investigated the effect of a grass forage diet, a legume forage diet and the more conventional forage and concentrate diet, on horses’ fluid balance and function of the hindgut fluid reservoir. The three diets implied differences in water intake, body weight and digesta water-holding capacity. Total water intake and body weight were higher when the horses were fed the lucerne forage diet but digesta water-holding capacity was higher when the horses were fed the young grass forage diet. It can be concluded that early harvested forage might benefit fluid balance of athletic horses without increasing body weight. In addition, further studies on plant-fibre and forage diets for horses are of great importance for horse feeding, for advisors, veterinarians and for the diet formulations industry.

**Abstract:**

Horses are herbivores, and their hindgut functions as a fluid reservoir as forage fibre properties have great impact on the water content of digesta and the milieu in the ecosystem. Our objective was to compare the effect of grass fibre maturity and legume forage on the water-holding capacity (WHC) and viscosity of the equine hindgut and the body weight (BW) and fluid balance of horses. Three diets: concentrate and late harvested grass haylage (35:65 energy ratio) (C); early and late harvested grass haylage (80:20) (G); lucerne and late harvested grass haylage (80:20) (L) were fed to six caecum and colon fistulated horses for 28 days in a Latin-square design. Total water intake and BW were higher when the horses were fed Diet L, but the digesta WHC was higher when fed Diet G. Total water excretion (via faeces + urine) and the difference in total water intake—output was higher when fed Diet L. Viscosity, measured on centrifuged digesta fluid, did not differ between diets, but the individual colon data of one horse were higher. In conclusion, early harvested forage might be beneficial for the fluid balance of athletic horses providing a higher WHC of hindgut digesta without increasing BW. The importance of digesta viscosity in relation to equine diets needs further investigations.

## 1. Introduction

Horses are large herbivorous hindgut fermenters, and the equine hindgut has been suggested to function as a fluid reservoir when dietary fibre holds water in the intestinal content [1,2,3]. The capacity of fibre to hold water is influenced by both the fibre structure and the chemistry of the liquid phase of the intestinal content. Fibre water-holding capacity (WHC) depends on both the variety and maturity of the fibre; lignin does not bind water at all [4,5]. Fibres high in WHC have been shown to ferment to a greater extent [6] and a greater volume of fluid available in the hindgut when more soluble fibre is included in the diet has been suggested [7]. Due to the greater fermentability of soluble fibre water would be made available as the fibre is being fermented in the hindgut. This would be the equine hindgut serving as a fluid reservoir to maintain fluid balance during dehydration [1].

Stage of maturity in grass forage has a negative impact on digestibility of dietary components [8,9]. The cell wall content increase as stage of maturity advances and hence the nutritive value of the forage decreases [9,10]. Increasing forage maturity influences the extent of fibre digestion and how dietary fibres act in the large intestine depends on the extent to which they are digested [11,12]. The effect of differences in grass forage maturity on the WHC of equine hindgut digesta and the fluid balance of the horse is not well investigated. In addition, grasses and legumes differ in both fibre content and fibre digestibility. Legumes contain less digestible but more lignified fibre than grasses [13] and large differences in ruminal degradation kinetics have been reported between grasses and legumes [14]. Legumes are high in pectin [15] which has high WHC but is also highly fermentable [6]. How differences between grass and legume forage affect the WHC of the equine hindgut and the fluid balance of the horse is not well investigated. To our knowledge there are no publications on in vivo measurements of WHC on equine digesta.

Fibre composition and diet regimens have been suggested to influence osmolality in the equine gastrointestinal tract affecting influx and absorption of water and electrolytes [16,17]. Previous osmolality measurements have shown a more hypertonic digesta on a conventional hay-grain diet and a hypotonic digesta on a low-protein, high-cellulose diet in equine caecum and right ventral colon after feeding [16]. The DM concentration of digesta in the caecum can be up to 5% higher on a concentrate diet compared to a roughage diet [18]. Because of this larger fluid and electrolyte reservoir in the hindgut high fibre diets may be desirable for high performing horses since exercise and competitions can imply large fluid losses [19]. Since the physical properties and chemical composition of forages have an impact on gut fill they can also affect the body weight (BW) [20] and an increased BW may be a disadvantage for the equine athlete [21]. However, recent studies have shown that changes in BW seem to depend more on type of forage than forage DM intake per se [22,23].

Viscosity is a physiochemical property associated with dietary fibres and it is particularly the soluble fibres that thicken when mixed with fluids [24]. It is well established, in humans, pigs, canines, rats and hamsters, that consumption of viscous fibres can increase the viscosity of digesta and for example lead to lower glycaemic and insulimic responses, and lower serum and liver cholesterol [24]. To evaluate viscosity in digesta is very difficult, it can vary considerably with sampling location, individual animal, gut motility and shear (force) rates of digesta within the gastrointestinal tract are not known [24,25]. To our knowledge there is no previous data published on viscosity of equine digesta fluid.

The aim of the present study was to compare the effect of feeding lucerne haylage, young grass haylage and the more conventional mature grass haylage and concentrate diet on the WHC, osmolality and viscosity of the equine hindgut digesta and the BW and fluid balance of the horse.

## 2. Materials and Methods

This experiment was conducted at AgroSup, Dijon, France, by approval of the Burgundy University ethical committee (agreement no. B0810).

### 2.1. Animals and Design

Six adult geldings (crossbreed, aged 7 to 15 years) were used, and they were fistulated in the caecum and the right ventral (RV) colon. Body weights ranged from 440 to 493 kg. Horses were kept in individual free stalls, had ad libitum access to water in buckets, access to a paddock with no grass and were exercised in a horse walker three times per week. The horses were randomised to the three diets in a Latin square design. The experimental periods were 28 days long and each started with a gradual shift in diet during the first 5 days.

### 2.2. Diets

The forages used were two grass haylages that were first cuts produced in the same field but harvested at different stages of maturity (6 weeks apart) and a lucerne haylage. Harvesting the grasses at two different stages of maturity resulted in different chemical composition, and in vitro digestible organic matter (IVDOM) values, metabolisable energy (ME) (Table 1 [26]).

The three diets were fed restrictedly to result in similar intakes of DM, ME and CP: the diet with concentrate (rolled oats, soybean meal) and late harvested grass haylage with a 35:65 energy ratio (Diet C), the grass diet with early harvested and late harvested grass haylage with an 80:20 energy ratio (+small amount of soybean meal) (Diet G) and the legume diet with lucerne haylage and late harvested grass haylage with an 80:20 energy ratio (Diet L) (Table 2). All diets were supplemented with a mineral product and salt.

Approximately 20% of the daily feed allowance was fed at 08.00 h, 20% at 10.00 h, 20% at 16.00 h and 40% at 17.30 h. The same ratios of concentrate:forage or forage:forage were fed at each meal. The diets, and feed analyses, are described in detail in an earlier publication [26].

### 2.3. Total Collection of Faeces and Urine, Caecum, Colon and Blood Sampling

At the end of each experimental period a 72 h total collection of faeces and urine was done with collection harnesses allowing a separate collection of faeces and urine. Collection harnesses were checked every hour and urine was emptied continuously and faeces every 12 h. During the total collection of faeces and urine, samples representing 24 h periods were prepared and frozen. In addition to the total collection of faeces and urine, samples for DM, WHC, viscosity and osmolality were collected (by gravity) via caecum and colon cannulas and grab samples were obtained from rectum. Samples were collected at 12.00 h, after the total collection of faeces and urine was finished. Blood samples were taken at 12.00 h at the end of each period, from one of the jugular veins by venipuncture, in heparinised tubes and centrifuged (1500× *g*, 10 min). The plasma was then frozen (−20 °C). The horses were weighed (Scale Tru-Test ACN076747174, Marechal Pesage, Chauny, France) at 09.00 h three times every week and daily during collection days at the end of each period. Horses had ad libitum access to water in buckets with litre measure. Water intake was measured and water refilled every 24 h during the 72 h of total collection of faeces and urine.

### 2.4. Digesta, Urine and Blood Analyses

Caecum and colon digesta and faecal samples were dried at 70 °C in an air-forced oven until constant weight and urine DM was obtained by drying in an air-forced oven for 24 h at 103 °C. WHC was measured with both the filtration and the centrifugation method [5,28] on dry digesta and faeces. For both the filtration and the centrifugation method 1 g of dry digesta or faeces was soaked in distilled water (350 mL) for 48 h. With the filtration method samples were filtered through Quantitative filter paper 454 (VWR European Cat. No. 516-0854, Avantor, Radnor, PA, USA) and left to drain for 30 min before obtaining wet weight. With the centrifugation method the wet samples were centrifuged at 3893× *g* for 20 min, excess water decanted off and the tubes left to drain for 30 min before obtaining wet weight. For both methods samples were dried in an air-forced oven at 103 °C over night before obtaining dry weight, then WHC was calculated as g water/g dry digesta or faeces. For viscosity and osmolality analyses caecum and colon content were filtered (squeezed) through a 100-μm Blutex nylon screen (SAATI, Sailly Saillisel, France). Samples for osmolality were frozen (−20 °C) and samples for viscosity kept on ice during transport to the laboratory and pending measurements. The viscosity was measured within 2 to 5 h after sampling on centrifuged (2000× *g*, 3 min, 4 °C) caecum and colon fluid supernatant with a Micro-Ubbelohde Viscometer (ISO 3105, 536 13/IC, Merck KGaA, Darmstadt, Germany). Osmolality of caecum and colon fluid supernatant (16,000× *g*, 5 min) was determined by freezing-point depression (Advanced Osmometer, Model 3250, Norwood, MA, USA). Total plasma proteins (TPP) were measured with a refractometer (Rogo-Sampaic, Wissous, France).

### 2.5. Statistical Analysis

Earlier experiments using four caecum and RV colon fistulated horses in change-over designs have proved sufficient for finding statistically significant differences examining the hindgut microbiota and its activity [29,30,31,32]. During the present experiment, one horse had to be excluded and therefore the statistical analysis was performed on five horses.

All variables were analysed by a statistical model including both fixed (period, diet, segment) and random (horse) effects. The model components were the overall mean, the effect of horse, the effect of period, the effect of segment, the effect of diet, the effect of the interaction between diet and segment, and the random error. All data were subjected to analysis of variance using the PROC MIXED of the SAS software (SAS Inst., Inc., Cary, NC, USA). Pair-wise *t*-tests were used to separate the main effect means and values are presented as least square means with the pooled standard error of the mean (SEM). Differences were considered statistically significant at *p* < 0.05. Pearson correlations (r) were calculated using PROC CORR of the SAS software (SAS Inst., Inc., Cary, NC, USA) and *p*-values were calculated for r = 0.

## 3. Results

Due to a colic on the concentrate diet, one horse had to be excluded from the experiment and therefore the statistical analysis was performed on five horses.

### 3.1. Body Weights and Fluid Balance

The horses were heavier when fed Diet L than Diet G and tended to be heavier when fed Diet L compared to Diet C (Diet C: 461 kg, Diet G: 459 kg, Diet L: 465 kg, SEM = 10.7, *p* = 0.025). Water intake by drinking did not differ between the diets, but the total water intake (drinking + water in feed) was greater when horses were fed Diet L than Diet C and G (Table 3). More water was excreted per day via the faeces when horses were fed Diet L compared to Diet G and the total water output (urine + faeces) was greater when fed Diet L compared to Diet C and G (Table 3). The difference in water intake—water output was greater when horses were fed Diet L compared to Diet C and G. The ratio of total water intake over total DM intake was greater when horses were fed Diet G and L than Diet C (Diet C: 3.6, Diet G: 4.5, Diet L: 4.4, SEM = 0.27, *p* = 0.022). The total water intake was not correlated to total ration DM, NDF and ADF intakes, but it was correlated to total forage DM and ADF intakes (Table 4). TPP did not differ between diets (Diet C: 69 g/L, Diet G: 69 g/L, Diet L: 69 g/L, SEM = 0.9, *p* = 0.875).

### 3.2. Physical Characteristics of Digesta

Using the filtration method, the WHC of digesta and faeces was greater when horses were fed Diet G than Diet C and L and higher in caecum than in colon and faeces but there was no interaction between diet and segment (Table 5). With the centrifugation method there was an interaction between diet and segment: in the caecum WHC was greater when horses were fed Diet G than Diet C with Diet L intermediate; in the colon WHC was greater when horses were fed Diet G than Diet C and L and when fed Diet G and L the WHC decreased from caecum to faeces (Table 5). Digesta DM concentrations were higher when the horses were fed Diet C compared to Diet G and L and between segments DM concentrations were higher in faeces but did not differ significantly between caecum and colon (Table 5). Viscosity of caecum and colon fluid did not differ between diets (Table 5), but the colon data for one horse were consistently higher than for the other four horses (1.46–1.93 vs. 1.02–1.26 mm^2^/s). Osmolality of caecum and colon fluid did not differ between diets or segments (Table 5).

## 4. Discussion

The aim of the present study was to compare the effect of feeding lucerne haylage, young grass haylage and the more conventional mature grass haylage and concentrate diet on the WHC, osmolality and viscosity of the equine hindgut digesta and the BW and fluid balance of the horse. The decrease in IVDOM from 89% to 63% reflects the increase in the stage of maturity of the grass haylage. The three diets resulted in small differences in DM and energy intake; 200 g/100 kg BW per day lower DM intake on Diet G compared to Diets C and L and 0.8 MJ/100 kg BW per day higher energy intake on Diet G compared to diet L. Diet L gave the highest and diet G the lowest intake of crude fibre, ADF, ADL and cellulose. In addition, Diet G provided lower NDF intake and Diet C highest and Diet L lowest intake of hemicellulose.

Increased forage intake has been shown to increase equine BW due to an increased total gut fill [20] and therefore also negatively affect exercise performance [33]. In contrast, more recent studies have demonstrated that the physical properties and chemical composition of forages have higher impact on BW than forage DM intake per se [22,23]. In the present study, DM intake was greater with Diet L than G (~0.9 kg/day) and horses were heavier when fed Diet L than Diet G. Despite significant, the difference in BW was small (6 kg) and might be explained with caution by a difference in gut fill [20]. Diet L provided the greatest intake of crude fibre, ADF, ADL and cellulose and maybe this diet gave in total the largest volume of undigested organic matter (‘bulk’) and water in the hindgut [23]. The DM and fibre intake was lower when the horses were fed Diet G but did not issue a difference in BW compared to Diet C. Diet G also resulted in the highest WHC of caecum and RV colon digesta. Maybe this lack of difference in BW between the forage and concentrate diet (Diet C) and forage-only diet (Diet G) might be due to the higher WHC of the caecum and RV colon digesta when horses were fed Diet G suggesting a larger amount of water in the hindgut. Total water content of the hindgut was not measured and the representativeness of digesta samples from cannulas regarding WHC of the digesta has to our knowledge not been investigated. However, we have measured higher WHC in faecal samples from exercising horses fed the same diets but 1.8 times maintenance intake [34] unpublished results. This may support the interpretation of the digesta WHC in the present study. Comparing measurements such as water intake, faecal pH and DM, plasma/blood urea between sedentary and athletic horses on the same diets have shown more apparent differences using athletic horses, probably because they are fed about twice the maintenance level [35].

A human study has reported an inverse relationship between the WHC of different fibres and their effect on faecal bulking of water, suggesting that dietary fibre does not affect faecal weight simply by retaining water in the gut [36]. The higher WHC of hindgut digesta when the horses were fed Diet G and the lack of difference in BW might suggest that higher hindgut WHC does not necessarily imply a heavier horse. Maybe with an early harvested grass, it would be possible to solve the contradiction between BW and forage intake for athletic horses.

Values of caecum and colon WHC in the present study were about 2.5-fold higher than previously reported values of rumen digesta [37] and more than four-fold higher than ileal digesta in grower pigs [38]. This might reflect the function of the equine hindgut as a fluid reservoir or simply differences in diet or gastrointestinal segment sampled.

In vitro studies have shown how fibres that hold more water ferment to a greater extent and fermentation directly reduces the WHC of fibre [6,39]. This is in accordance with the present study which resulted in significant decrease in WHC of digesta from caecum to faeces, which indicates that fermentation has decreased the WHC of the fibre fractions in the digesta as it passes through the large intestine. This is in accordance with fibre digestibility increasing along the hindgut from caecum to faeces [40]. In addition, the water concentration decreases from caecum to proximal and distal large colon [41] followed by the colonic separation mechanism assuring retention of fluid or transfer of DM to the small colon [42].

The two methods used to measure WHC of digesta, filtration and centrifugation, gave similar results but differences between diets were more pronounced with the centrifugation method and this might reflect some structural differences in the fibre fractions [5]. The centrifugation method gives an estimate of the water bound and trapped by the fibre but the susceptibility of the fibre to collapse under excess force during centrifugation makes the result difficult to interpret under physiological conditions and therefore the two methods should be considered together [5,28].

An in vitro study demonstrated that, although decreasing during digestion, the ranking of WHC between roughages was the same pre and post digestion and the roughage with the highest WHC also had the highest water releasing capacity [43]. The most suitable forage for the athletic horse would be the one with both the highest WHC but also the highest water releasing capacity, since that would imply the most available fluid reservoir. Water bound to fibre and excreted via faeces has not been available. Diet G comprised the most digestible forage and resulted in the highest WHC of hindgut digesta in combination with lower excretion of water via faeces. This is in accordance with the strong relationship between microbial digestion and the net movement of Na and water across the intestinal mucosa [44]. We have similar but more apparent results with exercising horses fed the same diets but 1.8 times maintenance intake [34] unpublished results. This would be the equine hindgut serving as a fluid reservoir to maintain fluid balance during dehydration [1]. Contradictory there was no difference in TPP between the three diets indicating that there was no effect on the plasma volume. However, blood was sampled 2 h after feeding and the post-feeding rise in TPP [23,45] might have overshadowed possible diet effects. Significantly lower TPP values have previously been shown on a forage-only diet compared to a forage-oats diet 5 to 12 h after fasting suggesting that plasma volume was better maintained during fasting on the forage-only diet [23]. An effect of diet has also been demonstrated on plasma protein concentration during exercise with lower TPP on a high (ad libitum) hay diet being indicative of greater water movement from the gastrointestinal tract into the plasma volume than on a limited hay diet [19]. Ponies fed a hay-grain diet showed hypertonic digesta up to 6 h after feeding which was associated with a net flux of water into the lumen and ponies fed a high-cellulose diet containing straw, alfalfa meal and urea showed hypotonic digesta 0–8 h after feeding with a net disappearance of water from the caecum and ventral colon [16]. The same study also showed large variation among individuals for liquid marker movement and intestinal water content [16]. In the present study, osmolality of caecum and right ventral colon fluid 2 h after feeding did not differ between diets or segments and was hypotonic to plasma (300 mOsm). A hypotonic colon fluid and a high individual variation in osmolality is in accordance with previous studies where forage-only diets were fed [29,30]. Diet C contained relatively small amount of concentrate and was intermediate in cellulose intake and probably not different enough to deviate in osmolality from Diet G and L. Perhaps large individual variations in water content and flow rates in the hindgut render individual variations in osmolality less surprising.

Water is continuously lost from the body via faeces and urine as well as via cutaneous and respiratory evaporation. The definition of water balance is when water losses and intake match and the BW is maintained [46]. The oxidation of feedstuffs also yields metabolic water. The oxidation of each gram of carbohydrate, fat, and protein yields about 0.6, 1.1 and 0.4 mL of water, respectively. For most domestic animals the metabolic water represents only 5 to 10% of the total water intake [47]. Calculated metabolic water for Diets L, G and C is approximately 0.6 to 0.9 L/day and 2 to 4% of total water intake. In the present study, fluid balance was measured as intake (drinking water + water content in the feed) versus output (faecal water + urinary water). The calculated evaporative fluid losses for sedentary horses of this size were reasonable estimates compared to literature [46]. The total water intake and total water output when horses were fed Diet L were larger compared to Diet C and G. Water intake in the equine has been correlated with DM intakes and the diet’s content of cell-wall constituents [48]. We found no correlation between the total water intake and the total ration DM, NDF or ADF intake, but total water intake was correlated to forage DM and forage ADF intake. This contrasts with athletic (1.8 times maintenance fed) horses offered the same three diets where total water intake was correlated to both total ration and forage DM, NDF and ADF intake [34] unpublished results. The ratio of total water intake over total DM intake was also higher when horses were fed Diet G and L compared to diet C which is in accordance with previous data where all forage diets gave a higher water to feed ratio than hay-grain diets [48].

The difference in water intake-output was also larger, ~1.1–1.2 kg/day, when the horses were fed Diet L compared to Diet C and G suggesting a higher evaporative loss on Diet L. The lucerne haylage had higher water content and provided more water via the feed. A previous study comparing hay and silage showed lower water intake by drinking, higher total water intake, higher estimated evaporative loss and higher digestibility when feeding silage [49]. It takes about 2.5 kJ to evaporate 1 g of water from the body [50], hence 1.3 kg/day corresponds to a heat loss of ~3.3 MJ. The higher estimated evaporative loss when the horses were fed Diet L might indicate a higher heat increment of feeding on this diet, which could be explained by the greater intake of crude fibre, ADF, ADL and cellulose with Diet L. This could have contributed to the higher total water intake when the horses were fed Diet L compared to Diet C and G.

The objective in the present study was not to achieve absolute values of equine digesta viscosity, but to perform a preliminary test of measurements when comparing three different diets. In the present study, a tube viscometer was used which has previously been used for measuring viscosities of rumen fluid [51] (personal communication) and pig caecal contents [52]. The viscosity of the centrifuged caecum and colon fluid did not differ between diets, but the intestinal fluid viscosity tended to be higher in the colon than the caecum, which might be logical as an increase in viscosity from caecum to rectum has been shown in pigs [53]. However, the higher mean viscosity values of the colon are predominately due to the individual values of one horse that had consistently higher colon values than the others.

Centrifugation of caecum and ileum samples from pigs and dogs have shown to result in substantial reductions in viscous characteristics of the digesta [52,54]. In this study the viscosity of the centrifuged hindgut fluid was not correlated to digesta DM concentration (data not shown) and using whole digesta might have given different results. Since viscosity is a physiochemical property associated with dietary fibres [24] measurements on whole digesta including more of the fibrous parts would probably come closer to physiological conditions. The importance of and approach for viscosity as a parameter for measuring changes in the hindgut of the horse needs further investigation. Health benefits of viscous fibres have been established in several mammals, lowering glycaemic and insulimic responses and serum and liver cholesterol [24]. It is particularly the soluble fibres that affect viscosity, and effects on plasma glucose and insulin have been reported when including soluble fibre in equine diets [55,56]. Assuming early harvested highly digestible forage include a larger portion of more fermentable fibre the effects on digesta viscosity and glucose and insulin responses would be of interest for further investigation.

## 5. Conclusions

In conclusion, the early harvested grass haylage diet resulted in higher caecum and RV colon WHC but not heavier horses. Forage harvested at an early stage of maturity might be beneficial for the fluid balance of athletic horses providing a greater WHC of hindgut digesta without increasing BW. The influence and importance of digesta viscosity in relation to equine diets need further investigations.

## Figures and Tables

**Table 1:** In vitro digestible organic matter (IVDOM), metabolisable energy (ME) and chemical composition, in g/kg DM if not otherwise stated, of the three forages used [26].

	Late Harvested Grass Haylage	Early Harvested Grass Haylage	Lucerne Haylage
IVDOM (%)	63	89	65
ME (MJ/kg DM)	7.5	11.6	8.7
Crude protein	80	172	157
Crude fibre	374	280	350
Neutral detergent fibre	670	521	483
Acid detergent fibre	409	299	382
Acid detergent lignin	50	22	77

**Table 2:** Intake of DM, energy, and dietary components of the three diets ^1^ in g/100 kg BW per day if not otherwise stated ^2^.

	Diet C	Diet G	Diet L	SEM	*p*-Values
DM (kg/100 kg BW per day)	1.4 ^a^	1.2 ^b^	1.4 ^a^	0.04	0.001
Energy (MJ/100 kg BW per day) ^3^	12.1 ^ab^	12.5 ^a^	11.7 ^b^	0.39	0.032
Crude protein	181 ^a^	199 ^b^	191 ^ab^	5.8	0.016
Crude fibre	420 ^a^	337 ^b^	499 ^c^	15.4	<0.001
Neutral detergent fibre	775 ^a^	617 ^b^	742 ^a^	23.3	0.001
Acid detergent fibre	465 ^a^	362 ^b^	544 ^c^	15.8	<0.001
Acid detergent lignin	54 ^a^	38 ^b^	97 ^c^	3.1	<0.001
Hemicellulose ^4^	309 ^a^	256 ^b^	197 ^c^	7.7	<0.001
Cellulose ^4^	407 ^a^	329 ^b^	446 ^c^	12.9	<0.001
Starch	91 ^a^	4 ^b^	16 ^c^	1.8	<0.001
Water soluble carbohydrates	61 ^a^	53 ^b^	21 ^c^	2.1	<0.001
Calcium	17 ^a^	18 ^b^	21 ^c^	0.6	<0.001
Phosphorus	3	3	3	0.1	0.235
Magnesium	3 ^a^	3 ^a^	5 ^b^	0.2	<0.001
Sodium	4 ^a^	4 ^b^	4 ^a^	0.1	0.010
Potassium	25 ^a^	33 ^b^	34 ^b^	1.3	0.001

^1^ Values are least square means with the pooled standard error of the mean. ^2^ Diet C: concentrate (oats, soybean meal) and late harvested grass haylage (35:65 energy ratio), Diet G: early and late harvested grass haylage (80:20) (+ small amount soybean meal), Diet L: lucerne haylage and late harvested grass haylage (80:20). ^3^ The estimated energy values, in mega joule, of the forages were calculated from the in vitro digestible organic matter values [27]. ^4^ Hemicellulose and cellulose concentrations in the feeds were calculated by weight difference: NDF-ADF and ADF-ADL, respectively. ^a,b,c^ Mean values within a row with unlike superscript letters were significantly different (*p* < 0.05).

**Table 3:** Daily water intake by drinking and via the feed and water output via faeces and urine (kg/day) in horses after three weeks of adaptation to forage based diets differing in fibre composition and maturity ^1,2^.

	Diet C	Diet G	Diet L	SEM	*p*-Values
	kg/Day				
Water intake	19.0	19.8	20.6	1.64	0.511
Water in feed	4.0 ^a^	4.0 ^a^	7.8 ^b^	0.11	<0.001
Total water intake ^3^	23.3 ^a^	23.6 ^a^	28.2 ^b^	1.69	0.020
Water in faeces	14.6 ^ab^	12.0 ^a^	17.1 ^b^	1.85	0.034
Water in urine	5.5	8.2	6.8	0.77	0.058
Total water output	20.0 ^a^	20.3 ^a^	23.8 ^b^	1.75	0.025
Difference intake-output	3.3 ^a^	3.2 ^a^	4.4 ^b^	0.30	0.043

^1^ Values are least square means with the pooled standard error of the mean (*n* = 5). ^2^ Diet C: concentrate (oats, soybean meal) and late harvested grass haylage (35:65 energy ratio), Diet G: early and late harvested grass haylage (80:20) (+small amount soybean meal), Diet L: lucerne haylage and late harvested grass haylage (80:20). ^3^ Water intake by drinking + water in feed. ^a,b^ Mean values within a row with unlike superscript letters were significantly different (*p* < 0.05).

**Table 4:** Pearson correlations between daily total water intake and daily intakes of DM, NDF and ADF from the total diet ^1^ or from the forage part of the diet in horses (*n* = 5).

	Total Water Intake	
	Correlation Coefficient	*p*-Values
Total DM intake	0.346	0.207
Total forage DM intake	0.648	0.009
Total NDF intake	0.213	0.446
Total forage NDF intake	0.480	0.070
Total ADF intake	0.502	0.057
Total forage ADF intake	0.583	0.023

NDF, neutral detergent fibre. ADF, acid detergent fibre. ^1^ Diet C: concentrate (oats, soybean meal) and late harvested grass haylage (35:65 energy ratio), Diet G: early and late harvested grass haylage (80:20) (+ small amount soybean meal), Diet L: lucerne haylage and late harvested grass haylage (80:20).

**Table 5:** Water-holding capacity (WHC) and DM of caecum and colon (right ventral) contents and faeces, and viscosity and osmolality of caecum and colon fluid in horses after three weeks of adaptation to forage based diets differing in fibre composition and maturity ^1,2^.

	Diet C	Diet G	Diet L	SEM	*p*-Values		
					Diet	Segment	Diet × Segment
**WHC filtration method (g H_2_O/g dry digesta)**							
Caecum	12.2	14.3	12.1	0.92	0.048	0.006	0.814
Colon	10.5	13.0	10.5				
Faeces	10.2	11.1	10.0				
**WHC centrifugation method (g H_2_O/g dry digesta)**							
Caecum	11.1 ^a^	14.2 ^bA^	12.4 ^abA^	1.04	0.012	0.005	0.044
Colon	10.7 ^a^	15.0 ^bA^	9.6 ^aB^				
Faeces	10.5	11.0 ^B^	10.0 ^B^				
**DM (%)**							
Caecum	5.7	2.4	4.2	0.74	0.043	<0.001	0.119
Colon	4.5	2.4	3.2				
Faeces	20.9	21.2	19.6				
**Viscosity (mm^2^/s)**							
Caecum	1.08	1.06	1.07	0.102	0.771	0.092	0.823
Colon	1.28	1.25	1.16				
**Osmolality (mOsm/kg of H_2_O)**							
Caecum	266	275	269	6.8	0.913	0.903	0.297
Colon	277	263	268				

^1^ Values are least square means with the pooled standard error of the mean (*n* = 5). ^2^ Diet C: concentrate (oats, soybean meal) and late harvested grass haylage (35:65 energy ratio), Diet G: early and late harvested grass haylage (80:20) (+ small amount soybean meal), Diet L: lucerne haylage and late harvested grass haylage (80:20). ^a,b^ Mean values within a row with unlike superscript letters were significantly different (*p* < 0.05). ^A,B^ Mean values within a parameter and column with unlike superscript letters were significantly different (*p* < 0.05).

## Data Availability

Data are available under request to the authors.
